# Is There an Association of Physical Activity with Brain Volume, Behavior, and Day-to-day Functioning? A Cross Sectional Design in Prodromal and Early Huntington Disease

**DOI:** 10.1371/currents.hd.cba6ea74972cf8412a73ce52eb018c1e

**Published:** 2016-03-17

**Authors:** McKenzie Wallace, Nancy Downing, Spencer Lourens, James Mills, Ji-in Kim, Jeffrey Long, Jane Paulsen, PREDICT-HD Investigators and Coordinators of the Huntington Study Group

**Affiliations:** Frances Payne Bolton School of Nursing, Case Western Reserve University, Iowa City, Iowa, USA; College of Nursing, The University of Iowa, Iowa City, Iowa, USA; School of Medicine, Indiana University Purdue University at Indianapolis, Indianapolis, Indiana, USA; Department on Psychiatry, University of Iowa, Iowa City, Iowa, USA; Department of Psychiatry, Carver College of Medicine, University of Iowa, Iowa City, Iowa, USA; Carver College of Medicine, University of Iowa, Iowa City, Iowa, USA; Carver College of Medicine, University of Iowa, Iowa City, Iowa, USA

## Abstract

Background: Huntington disease (HD) is a genetic neurodegenerative disease leading to progressive motor, cognitive, and behavioral decline. Subtle changes in these domains are detectable up to 15 years before a definitive motor diagnosis is made. This period, called prodromal HD, provides an opportunity to examine lifestyle behaviors that may impact disease progression. Theoretical Framework: Physical activity relates to decreased rates of brain atrophy and improved cognitive and day-to-day functioning in Alzheimer disease and healthy aging populations. Previous research has yielded mixed results regarding the impact of physical activity on disease progression in HD and paid little attention to the prodromal phase.

Methods: We conducted analyses of associations among current physical activity level, current and retrospective rate of change for hippocampus and striatum volume, and cognitive, motor, and day-to-day functioning variables. Participants were 48 gene-expanded cases with prodromal and early-diagnosed HD and 27 nongene-expanded control participants. Participants wore Fitbit Ultra activity monitors for three days and completed the self-reported International Physical Activity Questionnaire (IPAQ). Hippocampal and striatal white matter volumes were measured using magnetic resonance imaging. Cognitive tests included the Stroop Color and Word Test, and the Symbol Digit Modalities Test (SDMT). Motor function was assessed using the Unified Huntington’s Disease Rating Scale total motor score (TMS). Day-to-day functioning was measured using the World Health Organization Disability Assessment Schedule (WHODAS) version 2.0.

Results: Higher Fitbit activity scores were significantly related to better scores on the SDMT and WHODAS in case participants but not in controls. Fitbit activity scores tracked better with TMS scores in the group as a whole, though the association did not reach statistical significance in the case participants. Higher Fitbit activity scores related to less day-to-day functioning decline in retrospective slope analyses. Fitbit activity scores did not differ significantly between cases and controls.

Conclusions: This is the first known study examining the associations between activity level and imaging, motor, cognitive, and day-to-day functioning outcomes in prodromal and early HD. Preliminary results suggest physical activity positively correlates with improved cognitive and day-to-day functioning and possibly motor function in individuals in the prodromal and early phase of the condition.

## Introduction

Huntington disease (HD) is a rare neurological disease affecting 2.71 persons for every 100,000 persons worldwide^1^ that leads to progressive behavioral, cognitive, and motor decline. This autosomal dominant disorder is caused by an expansion of the cytosine-adenine-guanine (CAG) repeat, which codes for the huntingtin protein^2^. The huntingtin protein is expressed ubiquitously and its function remains unknown, but is thought to play a role in transcription regulation^3^. Onset of clinical manifestations typically occurs between ages 30 and 50^3^. CAG repeat length may explain approximately 50–70% of the variance in age of onset^4^^,^ 5 ^,^ 6, with a recent study reporting a correlation of 0.53 between CAG repeat length and age of diagnosis^7^. Genetic modifiers are likely to contribute to the wide variance in age of onset, and several potential genetic modifiers have been identified^8^^,^ 9 ^,^ 10. Nevertheless, the range of age of onset of motor features can vary widely at each CAG repeat length^6^^,^ 7 ^,^ 11 ^,^ 12. The range with the greatest variability in age of onset encompasses those with CAG repeats between 40 and 48, which accounts for 80% of the gene-expanded (i.e. CAG ≥36) population^7^^,^ 13 ^,^ 14. Wexler and colleagues^6^, controlling for CAG repeat length along with shared and nonshared environments, found that in almost 4000 individuals, factors other than genetic and heritable factors accounted for approximately 60% of the variance in age of onset. More recently, Lee and colleagues^15^ found that 30% of variance in age of onset could be related to factors besides CAG repeat length. Together, these data indicate a significant amount of variance related to disease onset may be associated with other modifying factors.

Lifestyle factors, such as diet, substance use, cognitive reserve, and physical activity might be related to age of HD onset^5^^,^ 16 ^,^ 17 ^,^ 18. Pathological mechanisms in HD that could be impacted by lifestyle include oxidative stress and inflammation, neuronal damage, altered DNA synthesis and repair, and epigenetic modifications^19^^,^ 20 ^,^ 21 ^,^ 22 ^,^ 23.

Several randomized controlled trials have shown that physical exercise and physical therapy interventions improve gait, balance, mood, and quality of life, as well as cognitive and day-to-day functioning in persons with diagnosed HD^24^^,^ 25 ^,^ 26 ^,^ 27 ^,^ 28 ^,^ 29. Data also indicate physical activity relates to improved cognitive performance, physical functioning, mood, and quality of life in persons with dementia and mild cognitive impairment^30^^,^ 31 ^,^ 32 ^,^ 33. Physical activity protects against brain volume loss in healthy aging adults, and those at risk for, or diagnosed with, neurodegenerative diseases, including those that affect areas of the brain affected by HD, such as the basal ganglia and hippocampus^25^^,^ 34 ^,^ 35 ^,^ 36. Putative protective effects of exercise on cognition include decreased neuroinflammation^37^, increased brain-derived neurotrophic factor (BDNF) activity^38^^,^ 39, induction of neurogenesis^40^, upregulation of heat shock proteins^41^, and reduction of stress-induced hormone activity^42^.

Despite data indicating positive effects of physical activity in diagnosed HD, the impact of physical activity on prodromal HD remains unknown. Interventions to delay onset of HD should ideally begin before onset of motor features^43^, by which time the neurodegeneration is often extensive^44^. In a retrospective study of 154 people with diagnosed HD, researchers compared physical, intellectual, and passive lifestyles (based on leisure activities, education, occupation, and domestic activity) and found that a passive lifestyle (i.e., low-intensity physical activity and high sedentary activity) related to earlier onset and predicted age of onset independent of CAG repeat length, although physical activity alone was not associated with age of onset^5^. Data from mouse models are equivocal with regard to whether physical activity can delay onset of HD manifestations, with some studies indicating wheel running from an early age delays motor onset^45^^,^ 46 while others have suggested the opposite^47^. A single human case study in prodromal HD indicated running may have accelerated HD progression in a marathon runner^48^. The limited data regarding the impact of physical exercise in prodromal HD reveals that understanding the effects of differing levels of physical activity in people with the HD gene expansion is incomplete. Our study is the first that examines the relationship between physical activity and brain volume, as well as cognitive, motor, and day-to-day functioning in a prodromal and early HD sample.

## Methods

**Participants**

The results in this article are from a subset of participants from the Neurobiological Predictors of Huntington’s Disease (PREDICT-HD) study, a longitudinal 32-site observational study that has followed over 1300 gene-expanded individuals and nongene-expanded control participants for over 12 years^7^^,^ 49 ^,^ 50 ^,^ 51. All participants traveling to the University of Iowa PREDICT-HD site between July 2012 and May 2013 were invited to participate in the substudy. The study procedure was explained to all participants, and all participants gave written informed consent. The study was done in accordance with the World Medical Association Declaration of Helsinki.

Participants included gene-expanded cases and nongene-expanded controls. Participants are considered gene-expanded cases when they have CAG repeat lengths greater than 36. Controls have tested negative for the HD gene expansion with CAG repeat lengths less than 35. All analyses compared outcomes of physical activity between cases and controls.

Participants in PREDICT-HD have been tested for the HD gene expansion and know their gene status, but do not exhibit motor features sufficient for an HD diagnosis upon enrollment into the study. However, some participants meet the Unified Huntington’s Disease Rating Scale (UHDRS) criteria for motor diagnosis during the course study participation and are at that time considered to have early stage HD. There were four such participants in our sample. Participants undergo a battery of cognitive, behavioral, and motor assessments, and MRI scans. Participants were excluded from participating if they had any of the following: clinical evidence of an unstable medical or psychiatric illness; alcohol or drug abuse within the past year; learning or developmental disability requiring special education; or history of another neurological condition.

**Activity monitoring**

We used Fitbit Ultra activity monitors (Fitbit, Inc., San Francisco) and the self-report International Physical Activity Questionnaire (IPAQ)^52^ to collect physical activity data from participants. The Fitbit Ultra (herein referred to as Fitbit) is a pedometer with a built-in accelerometer and altimeter that calculates steps taken, steps climbed, and distance in miles. In a validation study, Fitbit was shown to underestimate energy expenditure by approximately 15%, but compliance was higher than with other devices^53^. In order to facilitate compliance, the Fitbit was preferred due to the possibility of mildly impaired cognitive function in prodromal and early HD^54^. In a treadmill validation study, the Fitbit was shown to be as reliable as ActiGraph for step counts, which was the primary metric used for analysis^55^. A systematic review evaluating the reliability and validity of common activity tracking devices found that the Fitbit was highly correlated with laboratory-based studies while other studies found that at some speeds, the Fitbit underreported steps^56^. Given the exploratory nature of this study we proceeded with the Fitbit trackers.

Participants were shown how to use the Fitbit and were instructed to wear it clipped on to their waist during three self-defined “typical” days in a one-week period and to record dates of use on a record sheet. For example, participants who worked 40 hours per week were instructed to wear the Fitbit for two work days and one weekend day. If they participated in a regularly-scheduled physical activity (e.g. exercise class, recreation league sport, gym workout), they were instructed to wear the Fitbit tracker on a day that included this activity. Participants were instructed to put the Fitbit on immediately after waking up on selected days, and to remove it only when engaging in physical activities involving water, when showering, and when going to sleep for the night. For physical activity that involved water and required removing the Fitbit, participants were instructed to record their activity on the record sheet. They were informed that they did not have to include showering as water activity. Participants were instructed to mail back the Fitbit trackers and record sheets in the provided addressed-and-stamped envelopes. Activity data were downloaded to the web interface provided by Fitbit, Inc. Fitbit activity scores were calculated by combining the mean number of steps, miles, and stairs climbed over the three days to generate a total activity score for each participant.

The 7-item IPAQ^52^ is a self-report measure of physical activity that prompts participants to report the number of days in the past week they engaged in sitting, walking, and moderate or vigorous physical activity, and to specify the number of hours and minutes per day they spent in each activity. The IPAQ has demonstrated adequate reliability and validity, including good test-retest reliability (Spearman’s ρ = 0.8 within 8 days), and fair to moderate correlation (ρ = 0.30) between the IPAQ and accelerometer data^57^. Although there are some reported concerns about the reliability of the short form^58^, we decided to use the IPAQ because it is a short measure that creates minimal participant burden. While the Fitbit supplies data regarding the quantity of exercise a person participates in on a daily basis, the IPAQ provides the self-reported level of vigor of physical activity levels. Participants completed the IPAQ at home and returned it with the Fitbit trackers. We calculated IPAQ scores using the MET-minutes (MET = median metabolic equivalent) method for continuous IPAQ scores^59^. Essentially, each participant indicates (for each category: walking, moderate, and vigorous activities) the average number of days during which they engage in the specified activity, and how many hours each day they engage in said activity. These values are then multiplied together by a corresponding weight based on estimated MET. It should be noted that sitting time is not factored into the IPAQ variable analyzed below. The IPAQ and Fitbit participant scores were subsequently matched to the entire PREDICT-HD dataset for analysis.

**Outcome variables**

Outcomes of interest included variables consistently shown to be associated with disease progression in HD^7^^,^ 60 ^,^ 61 ^,^ 62 ^,^ 63 ^,^ 64 ^,^ 65. The striatum and hippocampus imaging was conducted using various 1.5 and 3T scanners based on study site. Volumes were analyzed using BRAINS image-processing software^66^.

Cognitive tests included the Symbol Digit Modalities Test (SDMT)^67^ and the Stroop Color and Word Test^68^. The SDMT is a timed test in which participants have 90 seconds to match digits with a provided symbol code. The score reflects the number of correct responses, with higher scores indicating better cognitive function. The Stroop Color and Word Test is made up of three timed tests in which participants (1) read color names printed in black ink; (2) identify the color of ink patches on a sheet; and (3) identify the color of ink that color words are printed in, including matching and non-matching word-color combinations. The final score refers to the number of correctly stated items for each test in 45 seconds, with higher scores indicating better cognitive function.

The UHDRS is a clinician-rated exam that generates a total motor score (TMS) from a battery of motor assessments (e.g., gait, involuntary movement, eye movement, dysarthria)^69^. Scores can range from 0–124, with higher scores indicating greater motor impairment. The 12-item World Health Organization Disability Assessment Schedule (WHODAS) version 2.0 is a test of global day-to-day functioning^70^ in which higher scores indicate worse functioning. We chose to include the companion-rated measure in addition to the participant-rated measure due to evidence of impaired reliability of self-report in the late prodromal period^63^.

**Analysis**

All analyses were performed using the statistical software program R (version 3.1.2). The overall goal of the analysis was to determine whether activity level is associated with progression in HD, and our exploratory aim was to examine whether being physically active may have a protective effect with respect to HD-associated changes. We calculated correlations between activity level (as measured by Fitbit and IPAQ) and cognitive, imaging, motor, and functional variables to assess whether activity level correlates with variables collected in PREDICT-HD. We also calculated correlations separately for gene-expanded and nongene-expanded individuals so as to assess whether the correlation structure changed by gene status. Next, we fitted linear mixed models (LMMs) using all available longitudinal data collected between 2002–2014 from PREDICT-HD to calculate progression indices for individuals in the subset with physical activity data. Progression indices were defined in terms of fixed and random slopes, so that each individual's progression index is equal to his or her estimated annual change in the variable under consideration. Random slopes were then correlated with activity data. Multiple testing was accounted for using the false discovery rate (FDR).

## Results

**Descriptive analyses**

For this sub study, 87 PREDICT-HD participants at the University of Iowa were invited to participate. Of these 87, one person declined to participate due to time concerns; one participant was ill the day of the study and withdrew; one participant did not return physical activity data; nine participants had incomplete data (four participants did not return IPAQ forms, four returned incomplete IPAQ forms, and six people returned Fitbits with no data on them); and one participant for which activity data were collected was removed from the analysis because that participant’s data were not contained in the latest data cut. Therefore, 75 participants with complete Fitbit and IPAQ data were used for these analyses. Table 1 shows participant demographic data. Most of the gene-expanded participants were in the prodromal stage. Four participants who completed the study had UHDRS diagnostic confidence level scores of 4, indicating that they had symptoms consistent with HD motor diagnosis. The only significant difference between cases and controls was mean CAG repeat length (F = 953.53, p < 0.001). For all other comparisons, p > 0.40.

**Table 1. Participant demographic variables:** DCL = Diagnostic confidence level

Variable	Case	Control
n	48	27
Baseline mean age (SD)	45.68 (13.60)	47.99 (10.47)
Baseline mean age (SD) prodromal only	45.33	–
Gender: female n (%)	32 (66.66)	19 (70.37)
Gender: male n (%)	16 (33.33)	8 (29.63)
Mean CAG repeat length (SD)	41.81 (2.09)	20.63 (3.86)
Mean years of education (SD)	15.23 (2.20)	15.56 (2.22)
DCL = 4	4	N/A

**Activity monitoring**

Most participants returned the Fitbits along with the Fitbit activity record sheet and IPAQ questionnaires. Four participants did not return Fitbit activity records with the Fitbits; however, we were able to use the data for analyses by extrapolating their activity data and dates of activity from the Fitbit website. In these cases, the three days with the most complete data were used. Seven participants recorded removing the Fitbits to shower or for water activities (e.g. 5 hours at a water park, 1 hour water volleyball, 40 minutes water aerobics, 1 hour kayaking). Several participants made notes on the record sheets to indicate atypical activity. These aberrations were either more sedentary than usual (e.g., long car rides, watching a football game, heavy rain inhibiting daily walk) or more active than usual (e.g., shoveling snow, long shopping trip, training for a marathon). Two participants recorded they removed the Fitbit during intensive exercise (an intensive video exercise program, a marathon training run) because the intensive exercise was not “typical” for them. We also encountered some technical issues with our physical activity monitoring, with one of the Fitbits recording implausible data (e.g., a mismatch between steps taken and miles tracked in a day or a mismatch in self-reported dates of activity and Fitbit recorded dates). The tracker with step/miles mismatch was retired as soon as the problem occurred. One Fitbit tracker was lost, and one was not returned.

Table 2 presents means, standard deviations, and ranges for variables in the analyses and shows that cases had worse performance than controls on SDMT and TMS at the 0.05 level. At the 0.10 level, daily functioning for cases was worse than controls, as rated by both participants and their companions. These findings indicate that even in the prodromal phase, cases displayed subtle differences in cognitive, motor, and day-to-day functioning. Cases self-reported higher physical activity levels on the IPAQ at the 0.10 level, although cases did not significantly differ from controls in physical activity levels recorded by the Fitbits.

**Table 2. Means, standard deviations, ranges for all variables:** *Note*: ~ corresponds to 0.05 < p < 0.10, + corresponds to 0.01 < p < 0.05SDMT = Symbol Digit Modalities Test; WHODAS = World Health Organization Disability Assessment Schedule 2.0; (p) = participant; (c) = companion; TMS = total motor score from the United Huntington’s Disease Rating Scale; IPAQ = International Physical Activity Questionnaire.

Variable	Case mean (SD)[range]	n	Control mean (SD)[range]	n
SDMT	50.80 (11.77)+ [25–76]	45	58.11 (8.88)[43–78]	27
WHODAS (p)	16.67 (6.36)~ [12–35]	40	13.48 (1.97)[12–19]	24
WHODAS (c)	15.97 (4.39)~[12–26]	39	13.73 (2.61)[12–21]	18
TMS	8.23 (11.65)+[0–45]	47	2.15 (3.17)[0–12]	27
IPAQ	3728 (3696)~[0–17788]	39	2051 (2131)[0–8088]	25
Fitbit	6781 (3587)[1414–16091]	48	7137 (2515)[3455–15054]	26

**Cross-sectional correlations**

Table 3 contains cross-sectional correlations. For the combined sample, activity level (as measured by the Fitbit) positively correlated with SDMT at the 0.10 level (r = 0.25, p = 0.056) and negatively correlated with the participant-rated WHODAS (r = -0.36, p = 0.009). Fitbit activity level negatively correlated with TMS at the 0.10 level (r = -0.23, p = 0.080). Fitbit also positively correlated with IPAQ activity levels (r = 0.41, p = 0.002). For the cases only sample, Fitbit activity level positively correlated with SDMT (r = 0.37, p = 0.034) and negatively correlated with participant-rated functional level on the WHODAS (r = -0.37, p = 0.047). Fitbit activity level positively correlated with IPAQ (r = 0.47, p = 0.009). No associations were significant in controls.

**Table 3. Cross-sectional correlation table:** *Note:* ~ corresponds to 0.05 < p < 0.10, + corresponds to 0.01 < p < 0.05, ++ corresponds to 0.001 < p < 0.01. SDMT = Symbol Digit Modalities Test; WHODAS = World Health Organization Disability Assessment Schedule 2.0; (p) = participant; (c) = companion; TMS = total motor score from the United Huntington’s Disease Rating Scale; IPAQ = International Physical Activity Questionnaire.

	Combined sample		Cases		Controls	
Outcome Variable	IPAQ	Fitbit	IPAQ	Fitbit	IPAQ	Fitbit
Stroop Word	-0.17	0.07	-0.11	0.05	-0.20	0.10
Stroop Color	-0.13	0.13	-0.05	0.14	-0.16	0.03
Stroop Interference	-0.11	0.10	-0.13	0.11	0.08	0.04
SDMT	-0.04	0.25~	-0.02	0.37+	0.12	-0.13
WHODAS (p)	-0.01	-0.36++	-0.08	-0.37+	-0.09	-0.35
WHODAS (c)	0.10	-0.20	0.03	-0.21	0.12	-0.15
TMS	0.18	-0.23~	0.10	-0.24	0.34	-0.15
Hippocampal volume	-0.20	-0.07	-0.18	-0.17	0.08	0.03
Striatal volume	-0.10	0.06	-0.03	-0.05	0.27	0.18
IPAQ	–	0.41++	–	0.47++	–	0.35
Fitbit	0.41++	–	0.47++	–	0.35~	–

**Scatterplots of Fitbit activity level with PREDICT-HD variables**

Figure 1 depicts scatterplots of all cross-sectional pairs of Fitbit activity levels with commonly analyzed PREDICT-HD variables, such as the Stroop Color and Word Test, SDMT, WHODAS, TMS, and hippocampal and striatal brain volumes. These plots suggest activity levels, as measured with the Fitbit, may be associated with important outcomes in HD, including cognition (SDMT) and daily functioning (WHODAS).

**Scatterplots of correlations between Fitbit activity scores and outcome variables.**
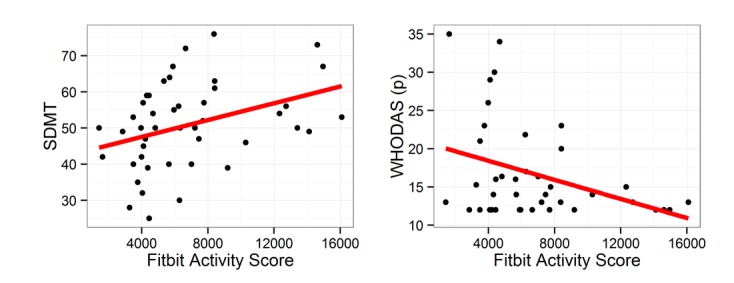
*Notes:* SDMT = Symbol Digit Modalities Test (higher scores indicate higher cognitive function); WHODASp = World Health Organization Disability Assessment Schedule 2.0 participant version (lower scores indicate higher day-to-day function).

**Random slope correlations**

A correlation matrix for the random slopes calculated from a linear mixed-effects regression analysis and activity level data is presented in Table 4. WHODAS participant slopes were negatively correlated with Fitbit activity level (r = -0.28, p = 0.039), and WHODAS (companion) slopes were negatively correlated with Fitbit activity level (r = -0.26, p = 0.065) at the 0.10 level.

**Table 4. Correlation matrices of random slopes and exercise indices:** *Note:* ~ corresponds to 0.05 < p < 0.10, + corresponds to 0.01 < p < 0.05. SDMT = Symbol Digit Modalities Test; WHODAS = World Health Organization Disability Assessment Schedule; (p) = participant; (c) = companion; TMS = total motor score from the United Huntington’s Disease Rating Scale; IPAQ = International Physical Activity Questionnaire.

	Stroop Interference	SDMT	WHODAS (p)	WHODAS (c)	TMS	Hippocampal Volume	Striatal Volume
IPAQ	-0.12	-0.10	-0.15	0.08	0.20	-0.06	-0.20
Fitbit	0.16	0.08	-0.28+	-0.26~	-0.20	-0.04	0.04

## Discussion

In this study, we examined associations between physical activity level and a variety of outcome variables with demonstrated sensitivity to changes in prodromal HD^7^. We collected physical activity data at participants’ most recent visit and compared this to their cognitive, behavioral, motor, functional, and imaging variables at the same visit, and to the rate of changes in the same variables from previous annual research visits. HD gene-expanded participants self-reported more physical activity on the IPAQ than nongene-expanded controls, but did not significantly differ in physical activity captured by Fitbit activity monitors. We used the short form of the IPAQ to limit participant burden. However, the short form of the IPAQ has questionable reliability^58^, which might account for the low correlations with the Fitbit scores in our study. The low correlations of the IPAQ to Fitbit scores may also be due to the fact that this is a cognitively impaired population, so perhaps self-reported exercise measures are not appropriate. Due to the limitations of the IPAQ, we focused on the associations between the more objectively recorded Fitbit physical activity measures rather than the self-reported IPAQ.

Given that Fitbit physical activity levels were similar between cases and controls, differences in outcome measures between cases and controls are less likely to be due to differences in level of physical activity between the groups. That is, any benefit seen is likely related to the level of physical activity since we only expect to see a cognitive or functional decline in the gene-expanded cases. In this study, higher Fitbit activity scores correlated with better cognitive functioning on the SDMT at the most recent visit for cases but not for controls. Since activity levels were similar between cases and controls this finding suggests that physical activity might provide cognitive benefits for people with the HD gene expansion.

The positive association between physical activity and the SDMT is a provocative preliminary result because the SDMT provides greater sensitivity to change in cognitive function in prodromal HD than the Stroop tests^71^. This enhanced effect size permitted us to identify changes in SDMT that would likely not be possible with Stroop tests. Our finding supports those from Piira and colleagues^28^, who found improvement on the SDMT, but no significant improvement on the Stroop tests, in a similarly-sized sample of early- and mid-stage HD participants following an intensive physical rehabilitation program.

We did not find evidence that physical activity is related to hippocampal or striatal volume, despite studies indicating that physical activity can maintain the volumes of these structures in aging adults and adults at risk for Alzheimer disease (AD)^34^^,^ 35 ^,^ 36. However, we did not collect longitudinal physical activity data, which is a limitation of this study and a necessity for future studies looking at brain volume changes over time. Physical activity may also produce cognitive benefits by promoting compensatory neuronal pathways via increased connectivity, which would require use of other imaging techniques, such as functional connectivity with resting state MRI or white matter tract integrity with diffusion tensor imaging.

In the rate-of-change analyses, we analyzed the correlations between physical activity, as measured at the most recent research visit, and rate of change over time. We found positive correlations between physical activity level and performance on the WHODAS, a measure of day-to-day functioning. This finding suggests physical activity might help preserve physical functioning and independence. However, we cannot discount the possibility that people who have better day-to-day functioning feel able to be more physically active. The relationship between higher activity levels and WHODAS performance is also a promising indicator that, if indeed physical activity can improve functioning and independence, health-related quality of life would also improve^63^.

Our study had a high recruitment-and-retention rate; only one PREDICT-HD participant declined to participate in the add-on study due to concerns about time commitment. In addition, 75/83 (90%) of the participants we enrolled completed the activity portion of the study, in line with another study involving an exercise intervention for people with diagnosed HD that had a retention rate of 81%^24^. This indicates that physical activity intervention programs are feasible, even in patients with early diagnosed HD.

This study had some limitations. We did not measure physical activity longitudinally. Instead, we made the assumption that one-time physical activity data for each participant would reflect their recent exercise history. However, collecting longitudinal physical activity data would be preferable. We have a small sample size for this study. More data are needed with larger samples to determine the effects of physical activity on cognitive function.

We also encountered some technical issues with the Fitbit, as noted above. More recent models of the Fitbit are now available and could provide more accurate data. It is also possible that people did not accurately report compliance with wearing the Fitbits. However, the number of people recording deviations in following the protocol (temporary removal) provides some indication that at least those participants were conscientious about using the Fitbit as instructed. There were other instances where a Fitbit was returned without self-reported date sheets. In these situations we extrapolated accurate data by using dates with physical activity recorded in the Fitbit database. There is also a limitation of the reliability of the accelerometer accuracy in a population that could have chorea movements. It is not known how this Fitbit device would calculate these disease-associated movements.

Also, we recruited participants year round. It is possible that participants recruited in the winter months had lower activity levels than others given that it is more difficult to participate in outdoor activities in cold weather. We also did not account for water activities that were not recorded by the Fitbit. In future studies, water activity should be accounted for, and all activity scores should be converted to one metric, such as METs. Despite the limitations in using the Fitbit and the IPAQ, our study demonstrates it is feasible to collect physical activity data from participants with prodromal and early stage HD. Future studies might also benefit from ensuring that a companion is involved, which might help ensure accurate completion of forms.

The prodromal participants in our study may be older than other prodromal individuals in the population. HD typically manifests in the late thirties or early forties. Our mean age of enrollment of 45.3 years for prodromal participants is comparable with manifest participants from other studies. If health behaviors impact age of onset, this age difference might reflect lifestyle factors that delay onset. For example, our gene-expanded participants had a higher average education level (15.1 years) than the average for United States residents. More education has been associated with better cognitive function in people with HD^72^, ostensibly by contributing to cognitive reserve, which is also associated with a reduced risk of AD^73^.

## Conclusion

Interventions that could delay the onset of an HD motor diagnosis, particularly at a time when people are at their peak earning potential and raising families, may improve functioning and health-related quality of life. Interventions should begin prior to motor diagnosis because there is some evidence that much of the damage the disease causes is done by the time of diagnosis^16^^,^ 74 ^,^ 75. More specific recommendations require prospective, randomized controlled trials of physical activity interventions. There is evidence that metabolic and physiological responses to exercise are altered in HD^76^ and that intensive exercise might damage muscle tissue^77^^,^ 78, requiring a careful determination of the proper dose of exercise to forestall HD progression. In one study involving aging adults at risk for mild cognitive impairment (MCI), moderate exercise was more strongly related to reduced odds for developing MCI than light or vigorous exercise^31^. Perhaps this is a recommendation that could be explored for relevance for HD.

Physical activity is an easy-to-implement, low-cost intervention that may help delay cognitive decline in gene-expanded persons, and also may have health benefits for at-risk HD family members who have not yet been tested or who do not want to be tested. Physical activity is a health behavior that can be started at a very young age. Children who are at risk can participate in family physical activities. Those habits started at a young age can continue throughout the child’s life, offering benefits for overall health, and could have a potential protective effect with respect to early cognitive decline associated with HD.

## Competing Interest Statement

Dr. Long has a consulting agreement with NeuroPhage, LLC, and is a paid consultant for Roche Pharma (F. Hoffmann-La Roche Ltd), and Azevan Pharmaceuticals, Inc.

Jane S. Paulsen has served on an advisory board for Lundbeck, LLC and has a consulting agreement with ProPhase, LLC.

## PREDICT-HD Investigators, Coordinators, Motor Raters, Cognitive Raters

Isabella De Soriano, Courtney Shadrick, and Amanda Miller (University of Iowa, Iowa City, Iowa, USA);

Edmond Chiu, Joy Preston, Anita Goh, Stephanie Antonopoulos, and Samantha Loi (St. Vincent’s Hospital, The University of Melbourne, Kew, Victoria, Australia);

Phyllis Chua and Angela Komiti (The University of Melbourne, Royal Melbourne Hospital, Melbourne, Victoria, Australia);

Lynn Raymond, Joji Decolongon, Mannie Fan, and Allison Coleman (University of British Columbia, Vancouver, British Columbia, Canada);

Christopher A. Ross, Mark Varvaris, Maryjane Ong, and Nadine Yoritomo (Johns Hopkins University, Baltimore, Maryland, USA);

William M. Mallonee and Greg Suter (Hereditary Neurological Disease Centre, Wichita, Kansas, USA);

Ali Samii, Emily P. Freney, and Alma Macaraeg (University of Washington and VA Puget Sound Health Care System, Seattle, Washington, USA);

Randi Jones, Cathy Wood-Siverio, and Stewart A. Factor (Emory University School of Medicine, Atlanta, Georgia, USA);

Roger A. Barker, Sarah Mason, and Natalie Valle Guzman (John van Geest Centre for Brain Repair, Cambridge, UK);

Elizabeth McCusker, Jane Griffith, Clement Loy, Jillian McMillan, and David Gunn (Westmead Hospital, Sydney, New South Wales, Australia);

Michael Orth, Sigurd Süβmuth, Katrin Barth, Sonja Trautmann, Daniela Schwenk, and Carolin Eschenbach (University of Ulm, Ulm, Germany);

Kimberly Quaid, Melissa Wesson, and Joanne Wojcieszek (Indiana University School of Medicine, Indianapolis, Indiana, USA);

Mark Guttman, Alanna Sheinberg, Albie Law, and Irita Karmalkar (Centre for Addiction and Mental Health, University of Toronto, Markham, Ontario, Canada);

Susan Perlman and Brian Clemente (UCLA Medical Center, Los Angeles, California, USA);

Michael D. Geschwind, Sharon Sha, Joseph Winer, and Gabriela Satris (University of California, San Francisco, San Francisco, California, USA);

Tom Warner and Maggie Burrows (National Hospital for Neurology and Neurosurgery, London, UK);

Anne Rosser, Kathy Price, and Sarah Hunt (Cardiff University, Cardiff, Wales, UK);

Frederick Marshall, Amy Chesire, Mary Wodarski, and Charlyne Hickey (University of Rochester, Rochester, New York, USA);

Peter Panegyres, Joseph Lee, Maria Tedesco, and Brenton Maxwell (Neurosciences Unit, Graylands, Selby-Lemnos & Special Care Health Services, Perth, Western Australia, Australia);

Joel Perlmutter, Stacey Barton, and Shineeka Smith (Washington University, St. Louis, Missouri, USA);

Zosia Miedzybrodzka, Daniela Rae, Vivien Vaughan, and Mariella D’Alessandro (Clinical Genetics Centre, Aberdeen, Scotland, UK);

David Craufurd, Judith Bek, and Elizabeth Howard (University of Manchester, Manchester, UK);

Pietro Mazzoni, Karen Marder, and Paula Wasserman (Columbia University Medical Center, New York, New York, USA);

Rajeev Kumar, Diane Erickson, Christina Reeves, and Breanna Nickels (Colorado Neurological Institute, Englewood, Colorado, USA);

Vicki Wheelock, Lisa Kjer, Amanda Martin, and Sarah Farias (University of California, Davis, Sacramento, California, USA);

Wayne Martin, Oksana Suchowersky, Pamela King, Marguerite Wieler, and Satwinder Sran (University of Alberta, Edmonton, Alberta, Canada);

Anwar Ahmed, Stephen Rao, Christine Reece, Alex Bura, and Lyla Mourany (Cleveland Clinic Foundation, Cleveland, Ohio, USA);

**Executive Committee**

Principal Investigator Jane S. Paulsen, Jeffrey D. Long, Hans J. Johnson, Thomas Brashers-Krug, Phil Danzer, Amanda Miller, H. Jeremy Bockholt, and Kelsey Montross.

**Scientific Consultants**

Deborah Harrington (University of California, San Diego); Holly Westervelt (Rhode Island Hospital/Alpert Medical School of Brown University); Elizabeth Aylward (Seattle Children’s Research Institute); Stephen Rao (Cleveland Clinic); David J. Moser, Janet Williams, Nancy Downing, Vincent A. Magnotta, Hans J. Johnson, Thomas Brashers-Krug, Jatin Vaidya, Daniel O’Leary, and Eun Young Kim (University of Iowa).

**Core Sections**

Biostatistics: Jeffrey D. Long, Ji-In Kim, Spencer Lourens (University of Iowa); Ying Zhang and Wenjing Lu (University of Indiana).

Ethics: Cheryl Erwin (Texas Tech University Health Sciences Center); Thomas Brashers-Krug, Janet Williams (University of Iowa); and Martha Nance (University of Minnesota).

Biomedical Informatics: H. Jeremy Bockholt, Jason Evans, and Roland Zschiegner (University of Iowa).
